# Perceptual learning shapes multisensory causal inference via two distinct mechanisms

**DOI:** 10.1038/srep24673

**Published:** 2016-04-19

**Authors:** David P. McGovern, Eugenie Roudaia, Fiona N. Newell, Neil W. Roach

**Affiliations:** 1 Trinity College Institute of Neuroscience and School of Psychology, Trinity College Dublin, College Green, Dublin 2, Ireland; 2 École d’Optométrie, Université de Montréal, Montréal, Québec, H3T 1P1, Canada; 3 Visual Neuroscience Group, School of Psychology, The University of Nottingham, Nottingham, NG7 2RD, United Kingdom

## Abstract

To accurately represent the environment, our brains must integrate sensory signals from a common source while segregating those from independent sources. A reasonable strategy for performing this task is to restrict integration to cues that coincide in space and time. However, because multisensory signals are subject to differential transmission and processing delays, the brain must retain a degree of tolerance for temporal discrepancies. Recent research suggests that the width of this ‘temporal binding window’ can be reduced through perceptual learning, however, little is known about the mechanisms underlying these experience-dependent effects. Here, in separate experiments, we measure the temporal and spatial binding windows of human participants before and after training on an audiovisual temporal discrimination task. We show that training leads to two distinct effects on multisensory integration in the form of (i) a specific narrowing of the temporal binding window that does not transfer to spatial binding and (ii) a general reduction in the magnitude of crossmodal interactions across all spatiotemporal disparities. These effects arise naturally from a Bayesian model of causal inference in which learning improves the precision of audiovisual timing estimation, whilst concomitantly decreasing the prior expectation that stimuli emanate from a common source.

To promote effective interaction with the environment, the brain combines information received from different sensory modalities. Integration of redundant cues relating to a common source can improve the precision of sensory estimates and help ensure that perception remains unified^1^,^2^. However, these benefits must be tempered against the costs of integrating cues arising from independent causes. Achieving a functional balance between multisensory integration and segregation requires a means of distinguishing between sensory signals relating to a single source from those relating to multiple external events^2^,^3^. A reasonable strategy for performing this task is to restrict integration to signals that coincide in space and time. However, it is important that the brain applies some tolerance to discrepancies, due to errors incurred during physical transmission and sensory processing. For instance, the relative timing of visual and auditory signals at source is contaminated by differences in the speed that light and sound travel through air, as well as differences in neural transduction latencies^4^,^5^,^6^.

The tolerance of multisensory integration to asynchrony is often described in terms of a ‘temporal binding window’. According to this view, sensory signals occurring within a certain temporal proximity are combined into a single multisensory percept, while those separated by longer time delays remain segregated. In healthy young adults, estimates of the extent of the window typically span several hundred milliseconds^7^,^8^,^9^, but evidence is accumulating that the binding window may be broader in older adults^10^,^11^,^12^ and individuals with a range of neurodevelopmental disorders [see^13^ for a review]. While considerable inter-subject variability has been reported^14^, the size of the window appears to be relatively robust to different measurement methods within individuals^15^ [though see^16^].

Motivated by these findings and the success of perceptual learning in improving unimodal sensory processing [see^17^ for a recent review], recent studies by Wallace and colleagues have investigated whether perceptual training can be used to manipulate the temporal binding window^9^,^18^,^19^. In their initial study, participants were trained to discriminate between synchronous and asynchronous audiovisual stimuli through the provision of trial-by-trial feedback on response accuracy^9^. The same task was used to measure the temporal binding window, defined as the range of stimulus onset asynchronies over which mean accuracy exceeded a criterion level. Comparison of results obtained before and after training indicated a significant narrowing of the temporal binding window. While these findings suggest that perceptual learning can alter multisensory processing, we do not yet have a coherent understanding of the mechanisms at work. Interpretation of this result is complicated by that fact that participants were trained on the same task that was used to measure the binding windows, making it difficult to distinguish generalisable changes in the balance between multisensory integration and segregation from more task-specific improvements.

Here we take a different approach to examining how perceptual training changes multisensory integration. While we retain a similar temporal discrimination training regime to Wallace and colleagues, in pre- and post-training sessions we ask participants to perform an auditory spatial localisation task while ignoring discordant visual stimuli. This stimulus arrangement gives rise to the classic ventriloquist effect^20^,^21^,^22^, in which the perceived location of the auditory stimulus is biased towards the visual stimulus. By manipulating the position of the visual stimulus and its timing relationship to the auditory target, we are able to map out learning-induced changes to both temporal and spatial binding windows. Our results reveal two distinct effects of temporal discrimination training: (i) a specific narrowing of the temporal (but not spatial) tuning of the ventriloquist effect and (ii) a general reduction in the magnitude of crossmodal interactions across all spatiotemporal discrepancies.

Our findings can be readily interpreted using quantitative models of cue combination based on Bayesian statistical inference. Models of cue integration incorporating statistically optimal weighting of available information have proved successful in accounting for situations in which discrepant sensory signals are fused into a single percept^1^,^2^,^23^. More recently, Bayesian observer models incorporating prior expectations for both single and multiple sources have allowed characterisation of the broad spectrum of interaction effects, from complete fusion to partial cue integration and complete segregation^3^,^24^,^25^,^26^. Here we extend this modelling approach to demonstrate that the dual effects of perceptual training we observe reflect an increase in the precision of audiovisual timing estimates, coupled with a decrease in the prior belief that stimuli relate to a common cause.

## Methods

### Participants

Twelve participants between the ages of 18 and 34 years old (mean age = 25 years old, 6 female) took part in the study. All were naive to the purposes of the study and gave written informed consent prior to their inclusion. Participants had self-reported normal hearing and normal or corrected-to-normal vision. Testing was carried out over 5 consecutive weekdays, comprising a pre-training session, three training sessions and a post-training session. All recruitment and experimental procedures were approved by the School of Psychology Research Ethics Committee, Trinity College Dublin in accordance with the principles of the Declaration of Helsinki.

### Stimuli

**Figure 1:**
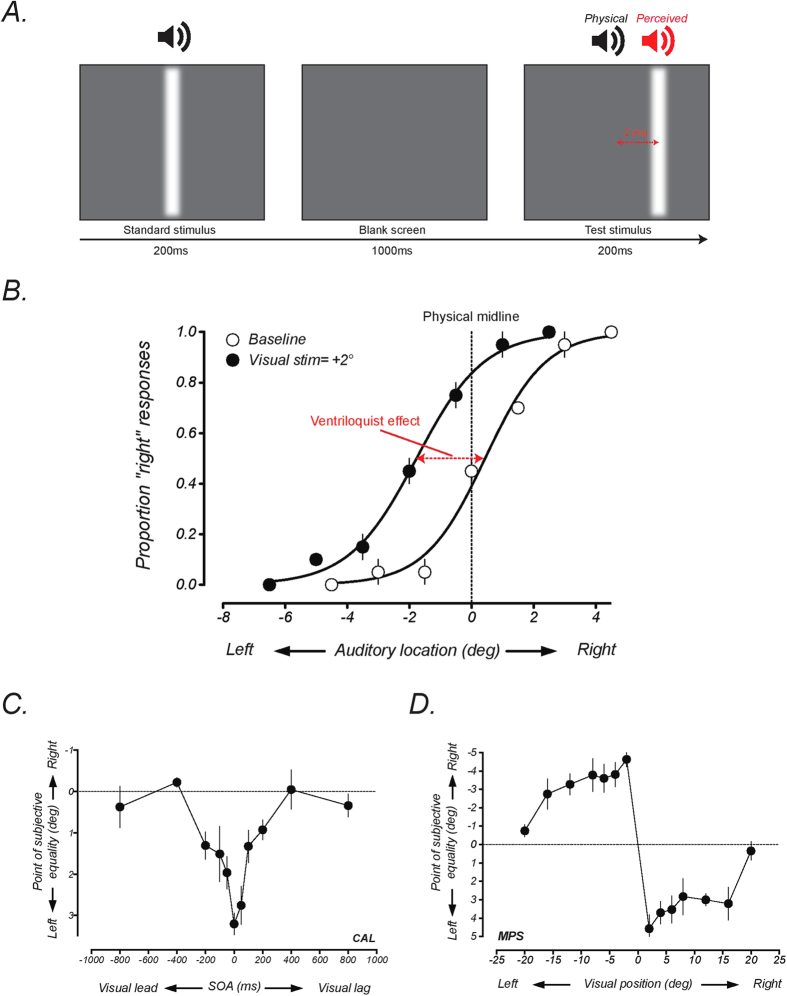
Schematic diagram of procedure used to measure temporal and spatial binding windows. (**A**) Participants were required to report whether an auditory noise burst in the second interval was to the left or right of a noise burst presented in the first interval. Auditory noise bursts were accompanied by visual bar stimuli masked with Gaussian luminance profiles. In the test interval, visual and auditory stimuli were presented in different locations leading to biases in the perceived location of the auditory stimulus. (**B**) For each condition, the magnitude of the ventriloquist effect was quantified by calculating the physical displacement of the auditory test stimulus required for it to be perceptually aligned to the standard stimulus. (**C**) Example of an individual temporal tuning function from Experiment 1 prior to training. The magnitude of the ventriloquist effect is maximal when auditory and visual stimuli were presented synchronously, and gradually declines with increasing asynchrony. (**D**) Example of an individual spatial tuning function from Experiment 2 prior to training. Ventriloquist effects were largest when the visual stimulus was presented close to the midline, but diminished at larger separations.

Visual stimuli consisted of vertical bars presented on a background of mean luminance (see Fig. 1A). The bars were full-screen height and had a horizontal Gaussian luminance profile with standard deviation of 2 deg. Visual stimuli were displayed on a gamma-corrected Dell Trinitron P1130 monitor at a resolution of 1024 × 768 pixels and a refresh rate of 60 Hz. At a viewing distance of 27.1 cm, each pixel subtended 5 arcmin of visual angle. Auditory stimuli consisted of bursts of bandpass-filtered (200 Hz-13 kHz passband) white noise presented binaurally via Sennheiser HD 250 headphones at a sound pressure level of 70 dB. Noise bursts were convolved with a non-individualised set of head-related transfer functions (HRTFs) containing both interaural time differences and spectral cues to localisation in azimuth [see^27^ for measurement details of HRTFs]. All stimuli were programmed in Matlab using functions from the Psychtoolbox^28^,^29^.

### Pre- and post-training sessions

Before and after training, participants performed variants of a two-interval forced-choice (2-IFC) spatial localisation task in which they discriminated the position of successive auditory noise bursts, while ignoring task-irrelevant visual stimuli (see Fig. 1A). In the standard interval, visual and auditory stimuli were presented synchronously and in spatial alignment. In the test interval, the location of the auditory stimulus was selected at random from 7 values via the method of constant stimuli, while the visual stimulus was positioned left or right of fixation at a set location (depending on experimental condition, see below). The presentation order of the standard and test intervals was randomised on each trial and participants were required to report whether the auditory stimulus in the first interval was to the left or to the right of the stimulus in the second interval. Auditory and visual stimuli were presented for 200 ms and the two intervals were separated by 1000 ms.

Psychometric functions were constructed describing the proportion of trials in which an individual observer judged the position of the auditory test stimulus to be positioned to the right of the auditory standard as a function of its position in azimuth (see Fig. 1B). Shifts in the perceived position of the auditory test stimulus were quantified by calculating the physical azimuth required to achieve perceptual alignment with the auditory standard (see Supplementary Methods).

For half of the participants (Experiment 1, n = 6), this approach was used to estimate the temporal binding window before and after training. Ventriloquist effects were measured using visual test stimuli positioned at 2 degrees to the right of fixation. Eleven different stimulus onset asynchronies (SOAs) were tested in separate runs (−800 ms, −400 ms, −200 ms, −100 ms, 0 ms, 100 ms, 200 ms, 400 ms, 800 ms), where positive and negative values indicate visual lag and visual lead conditions, respectively. For the remaining participants (Experiment 2), ventriloquist effects were measured for a range of visual test locations with synchronous auditory-visual presentation before and after training. Fourteen spatial positions were tested in separate runs, ranging from −20 (left of midline) to 20 degrees (right of midline). Examples of individual temporal and spatial tuning functions obtained prior to training are shown in Fig. 1C,D, respectively. Each participant completed 1–3 runs of 70 trials for each SOA or visual position condition in pre- and post-training sessions, yielding a minimum of 770 and 980 trials per each temporal and spatial tuning function, respectively.

In pre- and post-training sessions, participants also performed unimodal auditory and visual localisation tasks. The auditory localisation task consisted of a single interval forced-choice paradigm, where participants judged whether a stationary noise burst was positioned to the left or the right of the midline. Each noise burst was presented for 200 ms and its location was chosen at random from nine locations (range = 4 deg. to the left or right of the midline, step size = 1 deg.) centred around the midline via the method of constant stimuli. The visual localisation task consisted of a 2-IFC paradigm, where participants judged whether the position of a visual stimulus presented in the second interval was to the left or to the right of the stimulus presented in the first interval. The standard stimulus was presented at the midline and was equally likely to appear in the first or second interval. The position of the test stimulus was chosen at random from nine locations (range = 1 deg. to the left or right of the midline, step size = 0.25 deg.) centred around the midline via the method of constant stimuli. Both standard and test stimuli were presented for 200 ms and the two intervals were separated by 1000 ms.

### Training sessions

**Figure 2:**
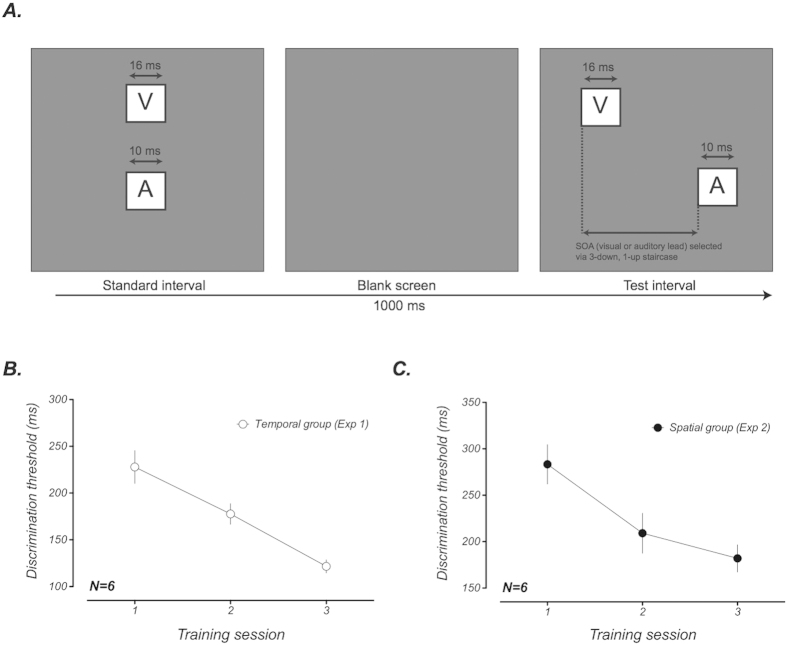
Schematic diagram of procedure for audiovisual simultaneity discrimination task and group-averaged learning curves. (**A**) Participants were required to discriminate whether a simultaneous audiovisual stimulus (the standard interval) was presented in the first or second interval. In the comparison interval, auditory and visual stimuli were separated by a stimulus onset asynchrony determined by a staircase procedure. Participants in both the temporal (**B**) and spatial (**C**) integration experiments improved on this task over the course of training. Error bars represent ± 1 standard error across participants.

In each training session, participants practiced a 2-IFC audiovisual simultaneity task, where they were required to judge which of two audiovisual pairs were presented synchronously. In the asynchronous interval, the absolute SOA between auditory and visual stimuli varied according to a 3-down, 1-up staircase (see Fig. 2A). In both intervals, the auditory stimuli were presented for 10 ms and the visual stimuli were presented for 16 ms (a single video frame) and all stimuli were presented at the midline. The order of the stimuli in the test interval (visual or auditory lead) and the presentation order of the synchronous and asynchronous intervals were chosen at random on each trial. The two intervals were separated by 1000 ms. Feedback was presented on a trial-by-trial basis, with high and low pitch tones indicating correct and incorrect answers, respectively. Discrimination thresholds were calculated as the mean of the last four reversals of the staircase and participants completed eight staircases of fifty trials in each training session (400 trials per training session). An average daily threshold for each participant was calculated as the mean of the eight threshold measurements.

### Data analysis

To quantify the effects of training on temporal and spatial binding windows, we fitted the group-averaged pre- and post-training data with a Gaussian function where the standard deviation and amplitude were left as free parameters. From these fits, we calculated learning ratios to summarise the changes in standard deviation and amplitude by dividing the best-fitting parameter values from the post-training data by the pre-training data, where learning ratios less than one indicated a reduction following training. To assess the statistical significance of any training-related changes, we carried out permutation tests on the Gaussian function fits by generating 10,000 resamples of the data, where the “pre” and “post” labels were randomly rearranged between different conditions. The same Gaussian function was fit to data from each of these resamples, creating a distribution of log learning ratios for both the standard deviation and amplitude under the null hypothesis. Two-tailed p-values were calculated as the proportion of sampled permutations in which the absolute log learning ratio was greater or equal to the equivalent value derived from the fits to the original dataset.

### Spatio-temporal causal inference model

To account for training-induced changes to the ventriloquist effect, we extended the Bayesian Causal Inference model^26^ to incorporate both the spatial position and timing of audiovisual signals. The generative model assumes a causal structure whereby audiovisual signals either relate to a single source (C = 1) or two independent sources (C = 2). These events are sampled from a binomial distribution with P(C = 1) = *p*_*common*_.

For a common source, auditory-visual signals are co-aligned in space. Their position *s*, is drawn from a broad prior Gaussian distribution centred straight ahead of the observer, such that *p*(*s* | *c* = 1) = *N*(*s*;*μ*_*prior*_ = 0 deg., *σ*_*prior*_ = 20 deg.) The SOA of the signals at the observer (*s*_Δ*t*_) is drawn from a relatively narrow distribution, centred on a slightly positive asynchrony to reflect the faster speed of light through air relative to sound^30^, *p*(*s*_Δ*t*_ | *c* = 1) = *N*(*s*_Δ*t*_*; μ*_Δ*t_common*_ = 40  ms., *σ*_Δ*t_common*_ = 50 ms). When there are two sources (C = 2), visual (*s*_*v*_) and auditory position (*s*_*a*_) are each independently drawn from the spatial prior distribution. The SOA is drawn from a broad Gaussian distribution *p(s*_Δ*t*_ | *c* = 2) = *N*(*s*_Δ*t*_*; μ*_Δ*t_ind*_ = 0 ms., *σ*_Δ*t_ind*_ = 800 ms). The observer’s noisy measurements of position (*x*_*v*_*, x*_*a*_) and SOA (*x*_Δ*t*_) are also normally distributed:


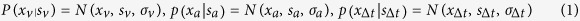


Assuming a squared loss function, the probability that a given audiovisual stimulus stems from a common cause can be inferred by combining the likelihood of the sensory measurements under a common cause with the prior probability, according to Bayes rule:


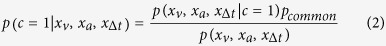


The likelihood of the sensory measurements given common and independent causes are given by:


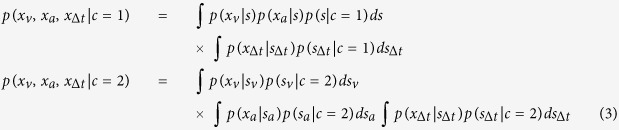


With only two possible causal structures, the probability of the sensory estimates is:


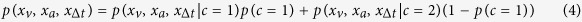


If the signals are known to have a common cause, the maximum-a-posteriori estimates of visual position (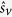), auditory position (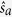) and SOA (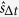) are given by:


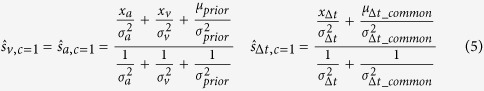


Whereas if the signals are known to relate to independent events, the maximum-a-posteriori estimates are:


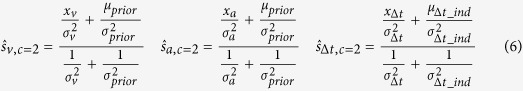


However, if the causal structure is unknown, the optimal solution is a weighted sum of these two conditional estimates:


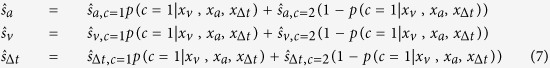


In simulations of the ventriloquist task, the model observer chose left on a trial if *ŝ*_*a*_ < 0, right if *ŝ*_*a*_ > 0 and produced a random guess if *ŝ*_*a*_ = 0. In the 2-IFC audiovisual simultaneity task, the model observer chose as simultaneous, the interval producing the smallest absolute SOA estimate |ŝ_Δ*t*_|.

## Results

### Training improves audiovisual temporal discrimination

In training sessions, participants practiced an audiovisual simultaneity task, where they had to report which of two sequentially presented intervals contained a simultaneous audiovisual stimulus (see Fig. 2A). Figures 2B,C show average discrimination thresholds for the two experimental groups across the three days of training. Consistent with previous studies [e.g.^9^,^18^], participants displayed an improvement in audiovisual simultaneity discrimination, with an approximate halving of discrimination thresholds following training. The improvement from the first to the third training session was statistically significant for both the temporal (*t*_(*5*)_ = 5.4, p = 0.003) and spatial (*t*_(*5*)_ = 3.6, p = 0.02) experimental groups. Although there was a trend suggesting that participants in the spatial group had higher thresholds overall than those in the temporal group, this difference was not statistically significant (*F*_(*1,30*)_ = 3.88, p=0.06).

### Experiment 1: Training reduces the width and amplitude of the temporal binding window

**Figure 3:**
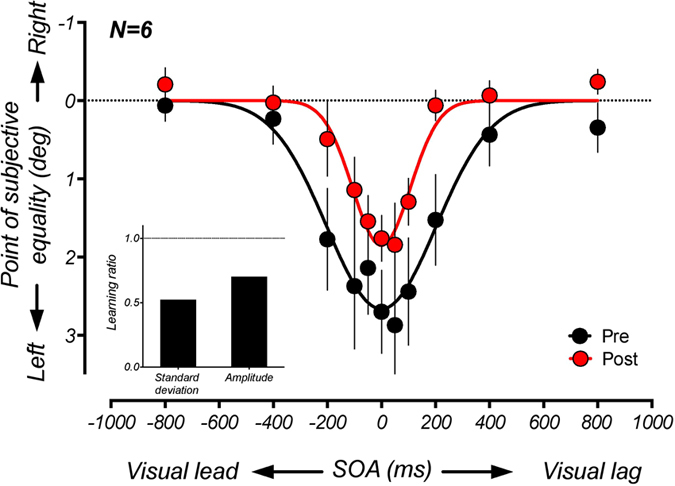
Group-averaged ventriloquist effects as a function of stimulus onset asynchrony (SOA) before and after training. Perceptual training caused a reduction in the magnitude of the ventriloquist effect across all SOAs. These reductions were particularly large for intermediate SOAs indicative of a narrowing of the temporal binding window. Changes between the pre- and post-training integration windows were quantified by fitting each dataset with a Gaussian function (R^2^ = 0.96 for both pre and post-training fits). The inset provides a summary of the changes to the standard deviation and amplitude of the Gaussian function following training. Data are expressed as learning ratios, calculated by dividing the post-training estimate of each parameter by the pre-training estimate. Both values are significantly less than one, indicating that these values are reduced in the post-training data. Error bars represent ± 1 standard error across participants.

For one group of participants, the ventriloquist task was used to estimate temporal binding windows before and after training. Figure 3 shows mean biases in the perceived position of auditory stimuli induced by a visual stimulus positioned 2 deg. to the right of the midline as a function of the relative timing of auditory and visual stimuli. Attractive shifts in the perceived auditory position towards the visual stimulus manifest as a systematic leftwards shift in the point of subjective equality. Both pre-training (black symbols) and post-training (red symbols) datasets exhibit clear temporal tuning, with the magnitude of this effect declining with increasing asynchrony. However, there is a marked reduction in the magnitude of the ventriloquist effect across all SOAs following training. These reductions were most prominent for intermediate SOAs (i.e ± 200 ms), consistent with the narrowing of the binding window reported in previous studies^9^,^19^.

To quantify these training-induced changes, we fitted the pre- and post-training data with Gaussian functions, leaving the standard deviation and amplitude as free parameters. The inset of Fig. 3 summarises changes in the best-fitting parameter values following training. Data are expressed as learning ratios (post/pre), whereby a value less than one indicates a reduction following training. In good agreement with previous findings [e.g.^9^], perceptual training led to a significant, near 50% reduction in the width of the integration window (learning ratio = 0.53, p = 0.006). However, our data also indicate a significant reduction in amplitude, illustrated by the change in the effect with synchronous stimulus presentation (learning ratio = 0.7, p = 0.004). To determine whether both of these components are strictly necessary to account for training-induced changes, we used the Akaike information criterion to compare these fits to those obtained with simpler models in which one parameter was shared between the datasets (see Supplementary methods). This analysis confirmed that the dual component fit outperformed the alternatives fits with a shared standard deviation (Δ*AICc* = *2.833*, *w*_*i*_ = 0.805) or amplitude (Δ*AICc* = *2.852*, *w*_*i*_ = 0.806).

### Experiment 2: Selective transfer of training effects to the spatial binding window

**Figure 4:**
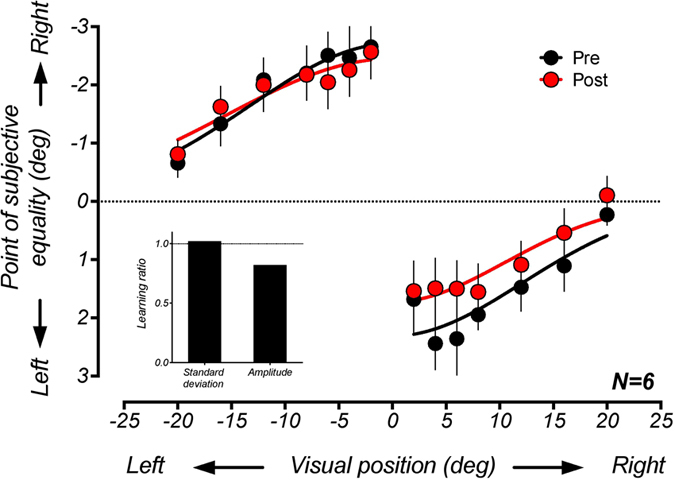
Group-averaged ventriloquist effects as a function of the position of the visual stimulus before and after training. Unlike the case of temporal integration, training did not appear to narrow the window of spatial integration. There was, however, an overall amplitude reduction of the ventriloquist effect, which was more pronounced for conditions where the visual stimulus was positioned to the right of the midline. To quantify this reduction in a similar manner to Experiment 1, separate Gaussian functions were fitted to the conditions where the visual stimulus was positioned to left and right of the midline (pre left: R^2=^0.96, post left: R^2^ = 0.89, pre right: R^2^ = 0.82, post right: R^2^ = 0.89). The best-fitting values for left and right conditions were then averaged to produce composite learning ratios for the standard deviation and amplitude, and these values are plotted in the inset of the figure. While there was a small decrease in the amplitude of the Gaussian fit following training, there was no change in the standard deviation (learning ratio = 1). Error bars represent ± 1 standard error across participants.

To investigate the specificity of changes in multisensory integration following audiovisual simultaneity training, we used the ventriloquist task to measure changes in the spatial binding window in a separate group of participants. Figure 4 summarises shifts in the perceived position of an auditory test stimulus presented synchronously with visual stimuli at different locations left and right of the midline. Points of subjective equality are consistent with the perceived auditory location being shifted towards the visual stimulus, with this effect declining in magnitude with increasing distance of the visual stimulus from midline.

Comparison of results obtained before and after training suggests that learning led to a reduction in the magnitude of the ventriloquist effect. Because of an apparent asymmetry in this effect, we fitted separate Gaussian functions for conditions where the stimulus was positioned to the left and right of the midline. Permutation tests revealed a significant reduction in amplitude for visual stimuli positioned on the right (learning ratio = 0.75, p = 0.04), but not on the left (learning ratio = 0.9, p = 0.33). In contrast, there was no significant change in the standard deviation on either side (left learning ratio = 1.16, p = 0.26; right learning ratio = 0.87, p = 0.33). To test the change in amplitude across all conditions, we also averaged the best-fitting values from left and right conditions prior to computing a single composite learning ratio (see inset of Fig. 4). This revealed that training led to a significant reduction in amplitude overall (learning ratio = 0.83, p = 0.03).

### Training-induced changes in multisensory integration reflect multiple mechanisms

**Figure 5:**
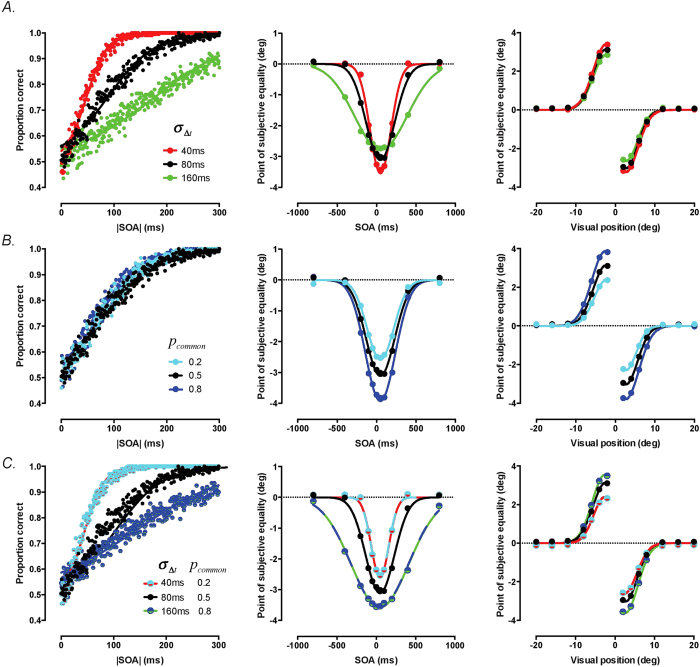
Accounting for the dual effects of training on audiovisual integration using a Bayesian causal inference model. (**A**) Improvement in the precision of SOA estimates explains narrowing of the temporal binding window, but predicts a concomitant increase in the magnitude of the ventriloquist effect with near-synchronous stimuli. Model simulations are shown for three levels of *σ*_Δ*t*_ on audiovisual simultaneity discrimination (left panel), the temporal tuning of the ventriloquist effect (middle panel) and the spatial tuning of the ventriloquist effect (right panel). (**B**) Changes in prior expectations explain the reduction in the amplitude of the ventriloquist effect. Model simulations are shown for the same three tasks, with different functions representing different levels of prior expectation about whether audiovisual stimuli relate to a common cause (*p*_*common*_). (**C**) Coupling reductions in both *σ*_Δ*t*_and *p*_*common*_ captures training-induced changes in performance across all three tasks. Unless otherwise indicated, simulations were carried out using the following parameter set: *σ*_Δ*t*_ = 80 ms, *σ*_*υ*_ = 1deg., *σ*_*a*_ = 2deg., *p*_*common*_ = 0.5.

To investigate the mechanisms through which perceptual learning alters multisensory integration, we simulated the effect of manipulating key parameters in a spatiotemporal causal inference model (see *Methods* for details). In each case, we carried out trial-by-trial simulations of task performance, replacing participant decisions with those of the model. Given that participants trained on a task requiring the discrimination of simultaneous and asynchronous audiovisual stimuli, the most obvious mechanism for driving changes in performance is an increase in the precision of their SOA estimates (i.e. reduction in variability). The left panel in Fig. 5A shows the effect of manipulating *σ*_Δ*t*_ in the model on the 2-IFC audiovisual simultaneity task performance. As expected, reducing *σ*_Δ*t*_produces a systematic improvement in response accuracy and reduction in SOA discrimination thresholds consistent with those shown during training. It is worth noting that the overall effect of this improvement is a steepening of the psychometric function relating accuracy to SOA. This change is therefore sufficient to explain previous reports of a narrowing of the temporal binding window [e.g.^9^], when quantified using this method.

Because the relative timing of audiovisual signals is an important cue for determining whether they relate to a common cause, changes in the precision of SOA measurements also has consequences for multisensory integration of spatial cues. Consistent with experimental data obtained in Experiment 1, reducing *σ*_Δ*t*_ narrows the temporal tuning of the ventriloquist effect (Fig. 5A, middle panel). This reflects improvement in the ability to infer whether or not a given SOA measurement may have been produced by a common cause. However, reducing *σ*_Δ*t*_ also results in an increase in the peak amplitude of the ventriloquist effect, the opposite of our findings. This occurs because an improvement in the precision of SOA measurements allows the model observer to infer with greater certainty that near synchronous audiovisual stimuli relate to a common cause. Since our spatial-tuning experiment was conducted with simultaneous stimuli, a general increase in the magnitude of the ventriloquist effect is also predicted for Experiment 2 (Fig. 5A, right panel). Clearly, learning-induced changes in the temporal bandwidth and amplitude of the ventriloquist effect must be produced by distinct mechanisms.

### Overall reduction in multisensory integration reflects changes in prior expectations

We reasoned that in practicing to discriminate synchronous and asynchronous audiovisual stimuli, observers may alter their prior beliefs regarding whether such stimuli relate to a common cause. Figure 5B shows that reduction of *p*_*common*_ has a negligible effect on audiovisual simultaneity task performance, as well as the temporal and spatial bandwidth of integration. However, by reducing the probability that any given audiovisual stimulus will be attributed to a common cause, it does scale the overall amplitude of the ventriloquist effect. Moreover by coupling reductions in *σ*_Δ*t*_ and *p*_*common*_, we are able to produce changes in performance across all three tasks that mimic those observed experimentally (Fig. 5C).

**Figure 6:**
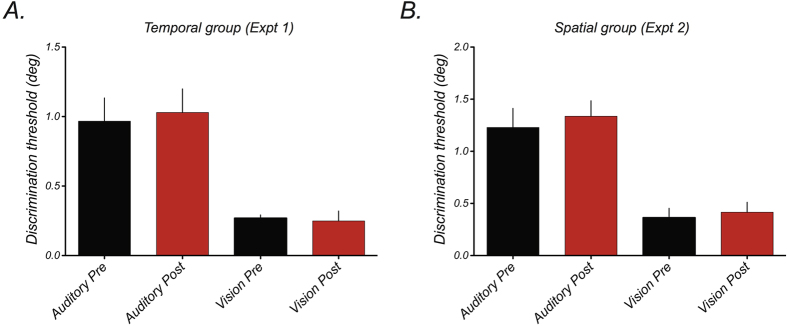
Unimodal positional discrimination thresholds measured before and after training. No changes in unimodal sensitivity were found following training for participants in either Experiment 1 (**A**) or Experiment 2 (**B**). Error bars represent ± 1 standard error across participants.

Prior expectation about causal structure is not the only factor that determines the overall magnitude of audiovisual interactions like the ventriloquist effect. In recent years, a large body of research has shown that these effects depend critically on the balance between unimodal sensitivities^2^,^23^,^31^. Under normal viewing conditions, the perceived position of auditory stimuli is strongly influenced by offset visual stimuli, due to the relative superiority of visual spatial resolution. However, this effect can be attenuated if the ability to localise visual stimuli is degraded^23^. Thus, an alternative explanation for the reduced ventriloquist effects following training might be an improvement in auditory spatial localisation and/or deterioration of visual spatial localisation. However, this prediction is not borne out in the data. As shown in Fig. 6, mean auditory and visual localisation thresholds do not differ before and after training for the two experimental groups (Temporal group, auditory: *t*_*(5)*_ = *0.30,* p = 0.77; visual: *t*_*(4)*_ = *0.31,* p = 0.77; Spatial group, auditory: *t*_*(5)*_ = *1.13,* p = 0.31; visual: *t*_*(4)*_ = *1.35,* p = 0.25). There were also no significant group-level differences in auditory or visual discrimination thresholds before or after training (Auditory pre: *t(*_*10*_) = 1.05, p = 0.31, Auditory post: *t(*_*10*_) = 1.36, p = 0.2; Visual pre: *t(*_*8*_) = 1.01, p = 0.34, Visual post: *t(*_*8*_) = 1.4, p = 0.2).

## Discussion

We have shown that perceptual training on an audiovisual temporal discrimination task produces two distinct effects on the integration of audiovisual input. The first effect is a specific narrowing of the temporal binding window, which does not generalise to spatial integration. The second effect is a general reduction in the magnitude of crossmodal interactions across all spatiotemporal discrepancies. To gain a better understanding of the mechanisms underlying these two effects, we simulated training-induced changes using an extension of the Bayesian causal inference model^3^,^26^,^30^. While models of this type have had considerable success in explaining the nonlinear effects of cue combination in source localisation^26^,^32^ and audiovisual speech^30^, to our knowledge this is the first study to apply this framework to characterise changes in multisensory integration caused by perceptual learning. Our analysis reveals that the dual effects of perceptual training we observe can be accounted for by an increase in the precision of audiovisual timing estimates together with a decrease in the prior belief that stimuli emanate from a common source.

Our finding that training improves audiovisual temporal discrimination is consistent with previous studies [e.g.^9^,^18^]. Moreover, since this same task was used to measure the extent of the temporal binding window in these studies, this improvement effectively replicates the training-induced narrowing reported (see Fig. 5A). By using a spatial localisation task to estimate the temporal binding window before and after training however, our study provides an additional test of the generality of these changes. Because training alters the range of asynchronies over which auditory and visual spatial cues interact, we are able to rule out the possibility that previous findings might be simply due to observers learning a strategy that is specific to the trained task. Rather, we can be confident that training induces a genuine shift in the balance between multisensory integration and segregation.

Changes in both audiovisual temporal discrimination and the tuning of the ventriloquist effect can be parsimoniously explained by an improvement in the precision of audiovisual timing estimates. According to this explanation, narrowing of the temporal binding window occurs because participants become better at inferring whether or not a given SOA measurement relates to a common cause.

It is important to note here that changes incurred via this mechanism can only be functionally adaptive. Even with extensive training and improvement, the width of the binding window will remain limited by the difference in temporal statistics of signals relating to common and independent sources. Thus, there is minimal risk that this form of training might result in a temporal binding window so narrow as to restrict the benefits of multisensory integration.

How does training improve the precision of audiovisual timing estimates? In principle, learning could affect temporal coding of auditory or visual signals, or alternatively target mechanisms that allow comparisons across sensory modalities^33^,^34^. Previous studies examining the transfer of training-related improvements between unimodal and multisensory timing tasks have produced mixed results. For example, whereas Stevenson *et al.*^19^ found that training on visual temporal order improved discrimination of audiovisual timing, Alais and Cass^35^ reported no transfer. Complicating the picture further, Alais and Cass found that audiovisual temporal order training improved visual temporal order judgements, but not auditory temporal order judgements. Clearly, more work is required to fully characterise the contributions of unisensory and multisensory processes to audiovisual training effects.

Our paradigm allowed us to reveal a previously unreported training effect on multisensory processing in the form of an overall reduction in the degree of multisensory integration. We can be confident that this reduction is distinct from the narrowing of the temporal binding window, since it is clearly evident even with synchronous audiovisual presentation (see Fig. 3). Moreover, our model simulations demonstrate that these effects must be driven by separate mechanisms. Whereas an improvement in the precision of SOA estimates is sufficient to explain narrowing of the temporal window, it actually predicts an increase in the magnitude of the ventriloquist effect obtained with synchronous stimuli (see Fig. 5A). Instead, we propose that the reduction is driven by a change in participants’ expectations regarding the causal relationship between audiovisual stimuli. Specifically, training participants to detect asynchronies between auditory and visual stimuli weakens the *a priori* belief that they relate to a common source (Fig. 5B).

It is generally assumed that prior expectations regarding causal structure vary according to the co-occurrence of sensory cues in the natural environment [e.g.^36^]. Auditory and visual signals are often correlated in space and time, promoting the expectation that any given pairing might relate to a common source. Despite being formed from a lifetime of experience, our results indicate that these expectations retain a degree of flexibility and can be altered with experience. These findings complement previous work showing that it is possible to learn to integrate sensory pairings that are normally unrelated in the world^37^. While it is likely that the repeated exposure to discrepant audiovisual stimuli during training was key to changing prior expectations, some important questions remain. For example, while previous research has shown that active engagement in audiovisual temporal discrimination is necessary to narrow the temporal binding window^9^,^19^, it remains unclear whether passive exposure might be sufficient to change prior expectations regarding causal structure. Further work is also required to establish the extent to which effects are specific to the characteristics of the trained stimuli. An unexplained finding from Experiment 2 was the asymmetric training effect observed when the visual stimulus was positioned on the right compared to the left. It remains to be seen whether this asymmetry is systematic and reproducible. One thing that is clear is that manipulation of prior beliefs is not dimension specific- note that our experimental design coupled training with temporally discrepant audiovisual signals with a task measuring biases induced by the integration of spatial cues.

In conclusion, we show that audiovisual temporal discrimination training leads to two distinct effects on multisensory integration in the form of a reduction in the width and amplitude of the temporal binding window. These effects are consistent with a Bayesian causal inference framework in which learning increases the precision of audiovisual timing estimates, whilst decreasing the prior belief that audiovisual stimuli originate from a common source. Coupled with recent progress in establishing how the brain accomplishes multisensory causal inference^38^, these results take us closer to a mechanistic understanding of how perceptual learning shapes the balance between multisensory integration and segregation.

## Additional Information

**How to cite this article**: McGovern, D. P. *et al.* Perceptual learning shapes multisensory causal inference via two distinct mechanisms. *Sci. Rep.* **6**, 24673; doi: 10.1038/srep24673 (2016).

## Supplementary Material

Supplementary Information
