# Supplementary material for: Perceptual learning shapes multisensory causal inference via two distinct mechanisms
Source: Sci Rep. 2016 Apr 19;6:24673. doi: 10.1038/srep24673 (PMC4835789; doi:10.1038/srep24673)
Supplement: Supplementary Information [file srep24673-s1.pdf]

Supplementary information: Perceptual learning shapes multisensory causal inference via two distinct mechanisms

David P. McGovern, Eugenie Rouadia, Fiona N. Newell and Neil W. Roach

Supplementary Experimental Procedures

Data fitting

To estimate the magnitude of the ventriloquist effect for a given condition, psychometric functions were constructed describing the proportion of trials in which an individual observer judged the position of the auditory test stimulus to be positioned to the right of the auditory standard (*p(test right)*) as a function of its position in azimuth (*X*). These functions were fitted with a logistic function of the form:

(S1)
$$p\,(test\ right) = \frac{1}{\left(1 + e^{\frac{PSA - X}{JND}}\right)}$$

where *PSA* is the point of subjective alignment and *JND* is an estimate of the participant's position discrimination threshold.

To characterise the tuning of the ventriloquist effect as a function of stimulus onset asynchrony (Expt 1) or visual stimulus position (Expt 2), group-averaged effects were fitted with a Gaussian function of the form:

(S2)
$$G(x) = a / \exp\left(0.5 \times \left(\frac{x - b}{c}\right)\right)$$

where *a, b,* and *c* are the amplitude, mean and standard deviation of the Gaussian fit, respectively. The best fitting values of *a* and *c* were taken as measures of the peak ventriloquist effect and the width of the binding window, respectively.

Gaussian fit model comparisons

Our data from *Experiment 1* suggests that perceptual training leads to a reduction in the bandwidth and the amplitude of the ventriloquist effect tuning function. However, it may be that changes to just one of these parameters could account for the data. For instance, the observed changes in the shape of the tuning function could potentially be explained by a general amplitude reduction in the ventriloquist effect across all SOAs, with little or no change in the width of the window (see *Figure S1* for schematic). To determine whether the best-fit values of the standard deviation and/or amplitude parameters differed between the pre- and post-training data, we calculated the second-order Akaike information criterion (AICc) for the Gaussian fit where all parameters were free to vary and for a "global fit" model where the parameter of interest was shared between the datasets. This analysis provides a trade-off between model complexity and goodness-of-fit, favouring a model with less parameters if the differences in the degrees of freedom outweigh the gains associated with a better model fit. The AICc for each of these models was calculated as follows:

(S3) $$AICc = N \times \ln(SS) + 2K * \left(\frac{N}{N - K - 1} \right)$$

where *N, SS* and *K* represent the sample size, sum-of-squares from the Gaussian fit and the number of free parameters in the model, respectively. The preferred model was chosen based on which produced the smallest overall AICc value. In the main text, we report the difference (*ΔAICc*) between the AICc estimates by subtracting the AICc value obtained from the independent Gaussian fit from the global fit value. Accordingly, positive *ΔAICc* values denote that the independent Gaussian fit was the preferred model, while negative values suggest the global fit model was preferred. To

assess the relative likelihood that a given model was correct, we also calculated weights based on the AICc values as follows:

(S4)
$$w_i = \frac{\exp(-0.5*(AIC_i - AIC_{\min}))}{\sum \exp(-0.5*(AIC_i - AIC_{\min}))}$$

where AIC_{min} and AIC_i represent the AICc values for the preferred and comparison models, respectively. These weights can be interpreted as the probability that each model is correct, with all probabilities summing to 1. For instance, when w_i is large (e.g. w_i=0.95 vs w_i=0.05) this indicates that one model is clearly preferred, however, when the difference in likelihood is small (e.g. w_i=0.51 vs w_i=0.49), this suggests that either model could be correct.

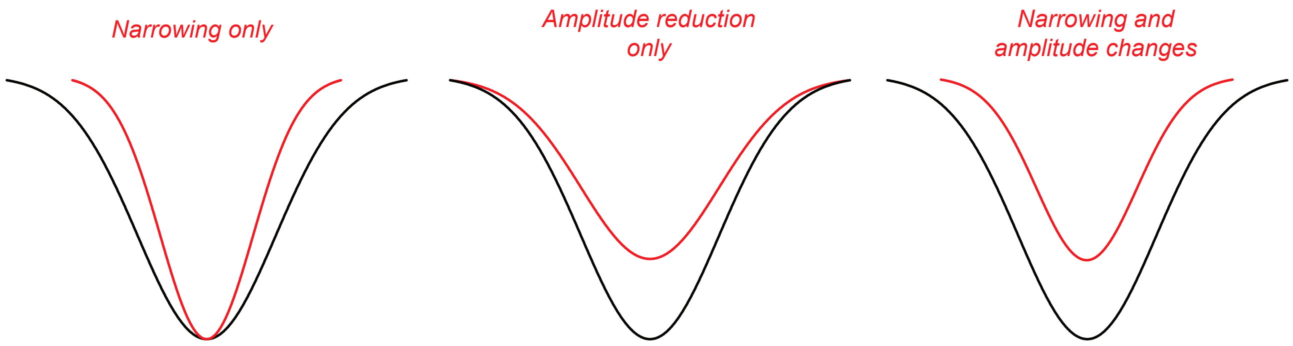

Supplementary Figure S1: Schematic representations illustrating how changes in the width alone, amplitude alone or changes to both would affect the shape of the temporal binding window following training.
